# Perception of Perspective Angles

**DOI:** 10.1177/2041669515593022

**Published:** 2015-07-30

**Authors:** Casper J. Erkelens

**Affiliations:** Helmholtz Institute, Utrecht University, The Netherlands

**Keywords:** visual space, perspective angles, models

## Abstract

We perceive perspective angles, that is, angles that have an orientation in depth, differently from what they are in physical space. Extreme examples are angles between rails of a railway line or between lane dividers of a long and straight road. In this study, subjects judged perspective angles between bars lying on the floor of the laboratory. Perspective angles were also estimated from pictures taken from the same point of view. Converging and diverging angles were judged to test three models of visual space. Four subjects evaluated the perspective angles by matching them to nonperspective angles, that is, angles between the legs of a compass oriented in the frontal plane. All subjects judged both converging and diverging angles larger than the physical angle and smaller than the angles in the proximal stimuli. A model of shallow visual space describes the results. According to the model, lines parallel to visual lines, vanishing at infinity in physical space, converge to visual lines in visual space. The perceived shape of perspective angles is incompatible with the perceived length and width of the bars. The results have significance for models of visual perception and practical implications for driving and flying in poor visibility conditions.

## Introduction

An extensive literature shows that linear perspective contributes to perception of depth and slant (Blake, Bülthoff, & Sheinberg, 1993; Braunstein & Payne, 1969; Cook, Hayashi, Amemiya, Suzuki, & Leumann, 2002; Erkelens, 2013a, 2013b; Flock, 1965; Freeman, 1965, 1966; Knill, 1998, 2007; Papathomas, 2002; Rogers & Gyani, 2010; Saunders & Backus, 2006; van Ee, Adams, & Mamassian, 2003). Linear perspective is a property of 2-D images and should not be confused with seeing perspective in 3-D scenes and objects. Euclid identified the latter type of perspective as natural perspective (Burton, 1945). There are hardly studies that address the question why we see perspective in 3-D scenes and physical objects. One reason for the lack of interest may be the assumption that natural perspective inevitably follows from seeing the world from one or two vantage points. A recent analysis of mechanisms underlying 3-D vision showed that natural perspective is not inevitable although 2-D images and thus retinal images are perspective projections of 3-D scenes (Erkelens, 2015). Natural perspective is manifest in vision because finite distances are assigned to vanishing points in the retinal images. Judgments of perspective angles between rails of a straight railway line indicated that distance assigned to vanishing points is extremely short (Erkelens, 2015). A second reason for little interest in natural perspective may be that in experiments 3-D objects are more difficult to present and manipulate than 2-D stimuli on a screen.

**Figure 1.**
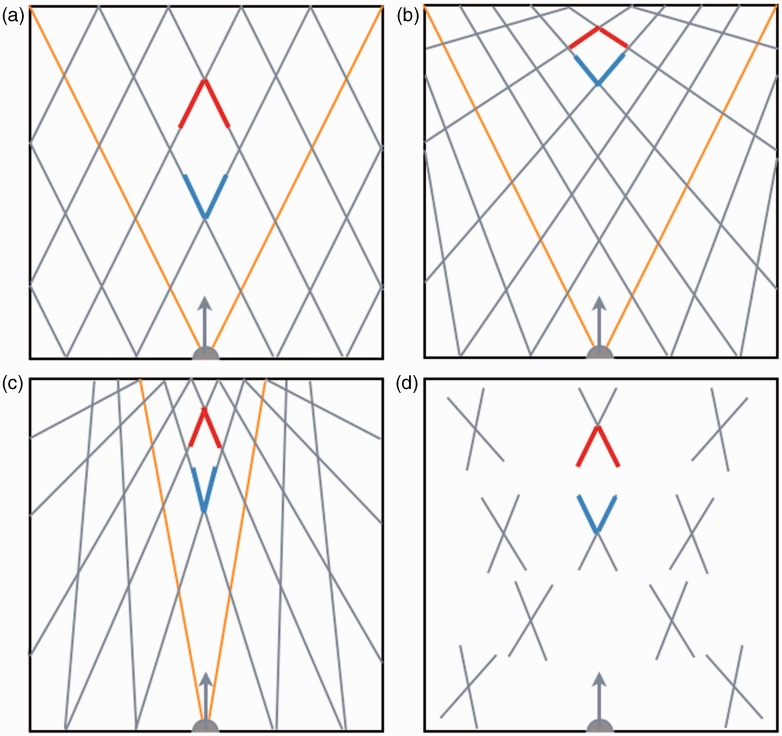
Geometries of spaces. The squares represent a large section of 2-D space in front of the head (gray half discs). Arrows indicate the viewing direction. (a) Physical space. Lines indicate visual directions (orange) and parallel lines (gray) that vanish at infinite distance. (b) Shallow visual space. Visual directions of (a) are unchanged but its parallel lines are rotated toward the visual directions. (c) Converged visual space. Visual directions and parallel lines of (a) are rotated toward the viewing direction. (d) Curved visual space. Crosses indicate rotated directions at several locations. Converging (red) and diverging (blue) angles in physical space (a) are larger in a shallow visual space (b), smaller in a converged visual space (c), and of equal size in a curved visual space (d).

Reported experimental results related to perceived visual directions and parallel lines make predictions for perspective angles, that is, angles that have an orientation in depth (Figure 1). From experiments in which observers constructed isosceles right triangles, Foley (1972) found that visual angles correspond closely to physical angles. Erkelens (2015) found that physically parallel rails appeared to make angles up to about 70° depending on the height of the eyes above the plane of the track. Together these results about perceived angles propose a visual space in which visual directions are identical to visual directions in physical space. Lines in parallel to visual directions in physical space converge to visual directions in visual space (Figure 1(b)). Another possibility is that all parallel lines in physical space converge to the viewing direction in visual space (Figure 1(c)). As a consequence, visual directions (“rays”) diverge less in visual than in physical space. Koenderink, van Doorn, de Ridder and Oomes (2010) claimed that visual directions in physical space are even parallel in visual space. A longstanding model of visual space is a curved space. The initial proposal of a Riemannian visual space (Luneburg, 1947, 1950) has been amended by many other authors (Blank, 1961; Cuijpers, Kappers, & Koenderink, 2000, 2002; Foley, 1972; Higashiyama, 1984; Indow, 1991; Koenderink, van Doorn, & Lappin, 2000; Musatov, 1976; Wagner, 1985). However, its alleged curvedness was not disputed. Curvedness means that visual directions are not straight so that the orientation of a straight line in physical space changes direction in visual space as a function of location (Figure 1(d)).

The three models of visual space make different predictions for the perception of angles between straight lines in physical space. Perspective angles, that is, angles oriented away and toward the observer, are wider in visual space than in physical space according to the shallow visual space model (Figure 1(b)). Oppositely, angles are smaller in visual space than in physical space according to the converged visual space model (Figure 1(c)). A property of a curved visual space is that deviations between visual and physical directions vary dependent on location. However, curved visual space is assumed to be locally Euclidean (Blank, 1953; Indow, 1991). This means that deviations affect all directions at one location so that angles are of equal size in physical and curved visual space (Figure 1(d)). In this study, subjects judged perspective angles between bars positioned on the floor of the laboratory to test the predictions of the three models of visual space.

## Experiment

### Stimuli

**Figure 2.**
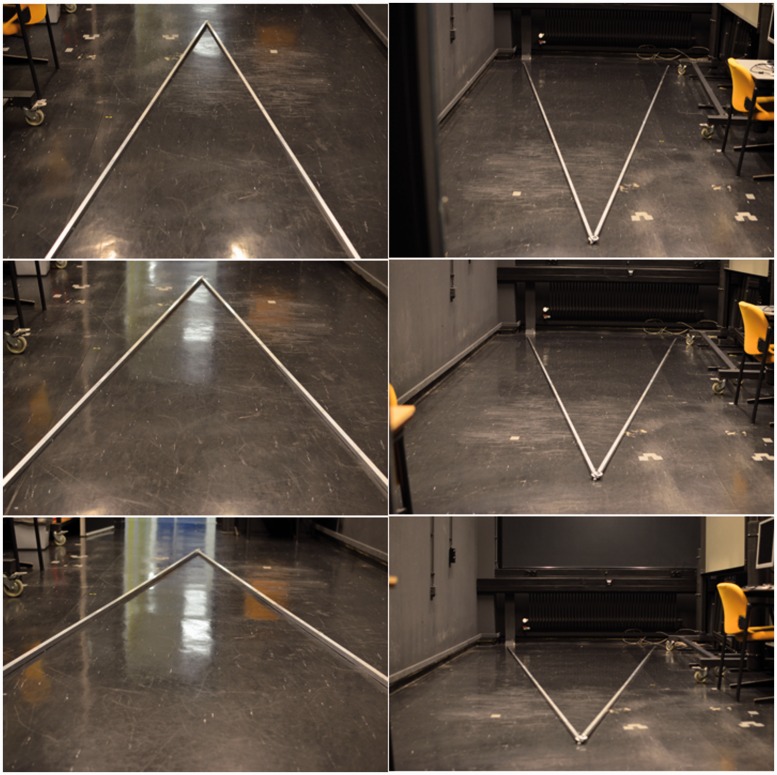
Stimuli. Pictures of converging bars (left figures) were taken from the three different eye heights used in the experiments. Right figures show similar pictures for diverging bars.

Two 5-m-long aluminum profiles (cross section 20 × 20 mm) were connected to each other at one end and placed on the floor of the laboratory (Figure 2). Distance between the other ends was 2 m. Angle between the bars was 23° in all experiments. For judgments of perspective angles between converging bars, observers were positioned at the center of the line between the proximal ends. For judgments of perspective angles between diverging bars, observers were positioned 3 m away from the apex.

### Experimental Setup

A pair of compasses was used to judge the perspective angles between the bars. Judgments were made at eye heights of 1.65, 1.15, and 0.75 m, respectively. From the same positions, pictures were taken with a Nikon D5100 camera fitted with a normal prime lens (Nikkor DX AF-S 35 mm f/1.8 G). A normal lens was chosen because it produces perspective in pictures that, if viewed from the correct distance, is natural to a human observer (Cooper, Piazza, & Banks, 2012). Field of view of the camera—lens combination was approximately 38° × 26°. The pictures were used to compare judged angles of physical and depicted bars mediated by similar proximal stimuli. The pictures were displayed on a TFT monitor (21″ LaCie 321, 1600 × 1200 pixels, 75 Hz). The screen measured approximately 43° × 28° at the viewing distance of 0.57 m. The pictures were projected at a size that was identical to the field of view of the camera—lens combination. A chin rest was used to fixate head position so that the center of the forehead (the “cyclopean eye”) was positioned at the center of projection of the pictures. The setup was placed in a normally lit room.

### Procedure

Four subjects (three physics students and the author) judged angles in the laboratory. The three students were experienced with judging angles in previous slant experiments but were naive with respect to the purpose of the study. The subjects had normal or corrected-to-normal vision and gave informed consent in accordance with the Declaration of Helsinki. The Ethics Committee of the Faculty of Social and Behavioural Sciences of Utrecht University approved the study. The approval is filed under number FETC14-018. To familiarize himself or herself with the setup, the subject was invited to walk up and down the room and to inspect the bars from various points of view. The bars were left untouched during the remainder of the experiments. The choice for a fixed and previewed angle between the bars was made to compare results with those of a recent study in which subjects judged perceived angles between a pair of long and parallel rails (Erkelens, 2015). To judge the angles at the three different eye heights, the subject had to stand, sit on a chair, and on the floor, respectively. The subject’s eye height was measured and adjusted when needed by using cushions before he or she made the judgments. For each measurement, the subject estimated the perspective angle between the bars, turned to the left or right, held the compass in a vertical position, and adjusted the angle between the legs until it was judged to match the remembered perspective angle. Turns of either head or torso of almost 90° to the left or right were made to prevent the subject from seeing bars and compass in a single view. The compass was held in a vertical position so that the perspective angle between the bars was matched to a nonperspective angle of the compass. The subject was allowed to repeat the procedure until he or she was satisfied with the result. The measurements were repeated 10 times during binocular viewing. The legs of the compass were closed after each measurement. The same measuring procedure was applied when subjects judged perspective angles in pictures on the screen.

## Results

**Figure 3.**
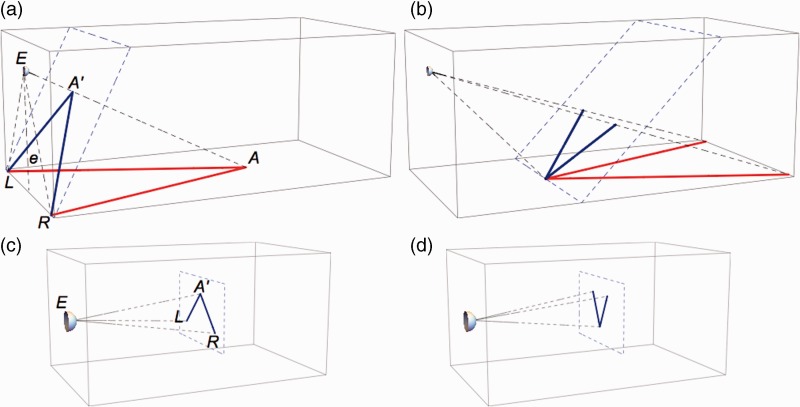
Stimulus geometry. Boxes show stimulus geometries for physical (a, b) and depicted (c, d) bars viewed from one of the three eye and camera heights (e). (a) The subjects viewed (*E* represents the position of the cyclopean eye) physical bars (red) converging at *A* on the floor. Angle *LA’R* (blue) indicates the proximal stimulus of the bars representing the retinal stimulus and the stimulus on the picture taken by the camera. The plane of the proximal stimulus is determined by the positions *L* and *R* of the near ends of the bars and an orientation perpendicular to *EA*, the visual direction of *A*. (b) Stimulus geometry for a subject viewing diverging bars. (c) The subject viewed the proximal stimulus *LA’R* (blue) on a frontal screen. (d) Stimulus geometry for the subject viewing pictures of diverging bars. For reasons of clarity, the dimensions of bars and eye height are not to scale.

For interpretation of the results, it is convenient to compare stimulus geometries for the physical and depicted bars (Figure 3). Figure 3(a) shows the geometry for an observer looking at converging bars lying on the floor of the laboratory. Angle *LAR* between the physical bars was 23° in all measurements. Proximal angle *LA’R* was computed from the physical positions of eye and bar ends. *LA’R* depended on eye height and was 65°, 83°, and 107° for the eye heights of 1.65, 1.15, and 0.75 m, respectively. For the diverging bars (Figure 3(b)), *LA’R* was 45°, 58°, and 79°. For the depicted bars (Figure 3(c) and (d)), angle *LA’R* was computed from the positions of *L*, *A’* and *R* on the screen. Ideally, proximal angles should have been identical for physical and depicted bars. However, differences up to 2° were observed probably due to small errors in camera positions. Apart from these small differences, proximal stimuli were identical for physical and depicted bars. On the other hand viewing distance, viewing direction and field of view were different. For the converging bars, the screen allowed vision of only the top parts of the bars (Figure 2).

**Figure 4.**
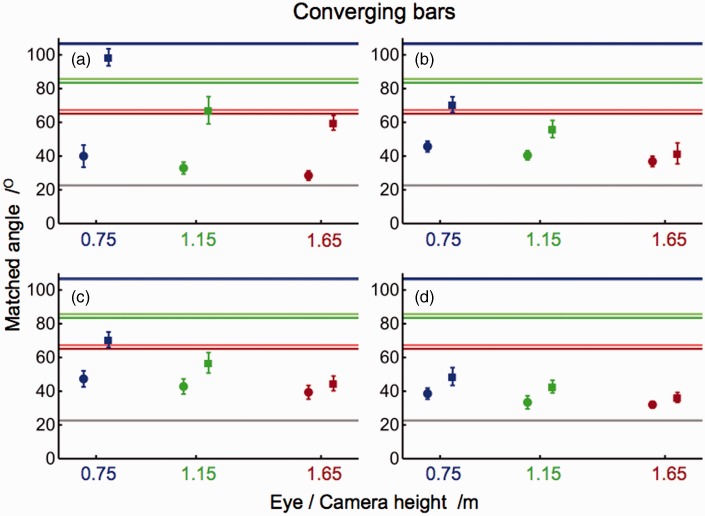
Matched angles of converging bars. Panels show means (±1 *SD*) of angles matched by subjects A, B, C, and D. Judgments were made for physical (dots) and depicted (squares) bars. Colored horizontal lines indicate proximal angles at eye and camera heights of 0.75 m (blue), 1.15 m (green), and 1.65 m (red). Proximal angles of physical (darker lines) and depicted (lighter lines) bars show differences due to small errors in the camera positions. Gray lines indicate the angle between the bars on the floor.

**Figure 5.**
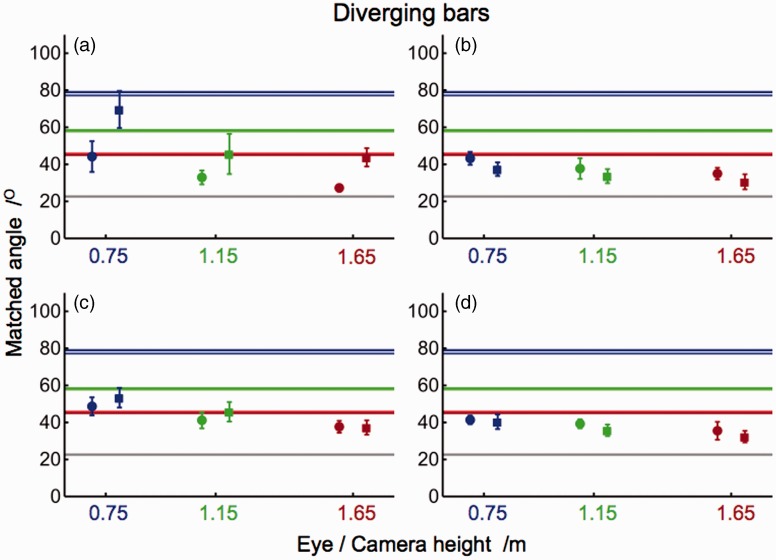
Matched angles of diverging bars. Panels show means (±1 *SD*) of angles matched by subjects A, B, C, and D. Judgments were made for physical (dots) and depicted (squares) bars. Colored horizontal lines indicate proximal angles at eye and camera heights of 0.75 m (blue), 1.15 m (green), and 1.65 m (red). Proximal angles of physical (darker lines) and depicted (lighter lines) bars may show differences due to small errors in the camera positions. Gray lines indicate the angle between the bars on the floor.

Figure 4 shows matched angles of converging bars for each individual observer as a function of height of eye or camera. Apart from a few outliers, individual data differed less than 10° from the means in most conditions and subjects. All subjects judged the perspective angles between physical bars smaller than those between bars in pictures taken from almost identical camera positions. All matched angles were smaller than the corresponding proximal angles and larger than the angle between the bars on the floor. Figure 5 shows the matched angles of diverging bars. In general, the results were similar to those of converging bars. Again, matched angles ranged between the physical bars’ angle and the proximal angle, except for a few judgments of subject A that were slightly larger than the proximal angle. In general, matched angles of depicted bars were larger than those of physical bars although differences were small in three of the four subjects. Sizes of matched angles showed a negative slope as a function of eye and camera height. Individual differences were predominantly observed in the matched angles of depicted bars. The mean of the judged perspective angles, averaged across all subjects and conditions, was 44° ± 7° and thus about twice as large as the physical angle of 23° between the bars on the floor.

**Figure 6.**
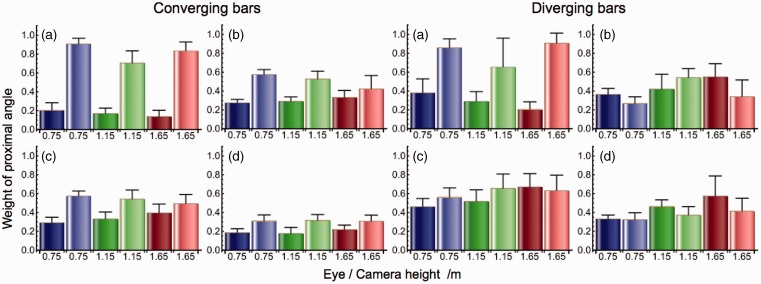
Weights of the proximal angle in the matched angles. Panels show mean weights (+1 *SD)* for angles matched by subjects A, B, C, and D. Judgments were made of angles between physical (darker colored bars) and depicted (lighter colored bars) bars.

The matched angles were expressed as weighted averages of the proximal and physical angles. Weights of the proximal angle in the judgments, computed as *w = (matched angle–physical angle)/(proximal angle–physical angle)*, are presented in Figure 6. Weights of the physical angle are equal to *1–w*. The results were analyzed in this way to test the hypothesis that perspective angles were perceived as a weighted average of the proximal and physical angles between the bars. Mean weight of the proximal angle, averaged across all subjects and conditions, was *w* = 0.44 ± 0.20. A four-way analysis of variance showed main effects of subject (*F_3,476_* = 28.5, *p* < .01), type of angle (converging vs. diverging; means 0.40 vs. 0.48; *F_1,478_* = 21.2, *p* < .01), and type of stimulus (physical vs. depicted; means 0.34 vs. 0.53; *F_1,478_* = 134.3, *p* < .01). The effect of eye and camera height (*F_2,477_* = 1.98, *p* = .14) did not reach significance. In view of considerable individual differences, three-way repeated measures ANOVA’s were also performed on the data of individual subjects. The factor type of angle was significant in subjects C (*F_1,118_* = 56.9, *p* < .01) and D (*F_1,118_* = 102.1, *p* < .01) but not in subjects A (*F_1,118_* = 1.00, *p* = .32) and B (*F_1,118_* = 1.74, *p* = .19). The factor type of stimulus was highly significant in subjects A (*F_1,118_* = 764.3, *p* < .01) and C (*F_1,118_* = 42.5, *p* < .01) but not in subjects B (*F_1,118_* = 2.92, *p* = .09) and D (*F_1,118_* = 0.51, *p* = .48). Height of eye and camera reached just significance in subjects C (*F_2,117_* = 3.45, *p* = .04) and D (*F_2,117_* = 6.30, *p* = .02) at a 5% criterion. Its effect was not significant in subjects A (*F_2,117_* = 1.90, *p* = .15) and B (*F_2,117_* = 1.32, *p* = .27). The fact that eye height hardly affected the weights supports the hypothesis that perspective angles are perceived as weighted averages of proximal and physical angles.

## Discussion

### Main Conclusions

Judgments of perspective angles were reproducible and consistent across the four subjects. Perspective angles were judged larger than the physical angle between the bars and smaller than its sizes in the proximal stimuli. Qualitatively, the model of a shallow visual space predicted perspective angles that were larger than the physical angle, while the other models did not (Figure 1). Quantitative differences were found between converging and diverging angles and between physical and depicted bars, differences that require an explanation. The model of a shallow visual space explains the differences between judgments of converging and diverging angles. The model predicts that deviations between visual and physical angles are larger at longer distance from the observer (Figure 1(b)). The vertex of the converging bars was located 5 m away from the observer, while the vertex of the diverging bars was positioned at a distance of just 3 m. Differences between physical and depicted bars may be explained by contributions of various cues to slant and depth that support each other in case of physical bars and oppose each other in case of depicted bars (Erkelens, 2015). Quantitatively, judgments of perspective angles were well described by a weighted average of physical and proximal angles. Weighted averaging of these angles is compatible with the shallow visual space model. A weighting factor of one of the proximal angle is equivalent with a vanishing point at zero distance and thus absence of depth. A weighting factor of zero is equivalent with a vanishing point at infinity in which case visual space is identical to physical space. An intriguing question is whether the weighting factors would have been different for angles that were not previewed by the observers. The current choice for previewing allows comparison of weighting factors of one subject, the author, with weighting factors measured in a previous experiment where subjects judged perspective angles between rails (Erkelens, 2015). His mean weighting factor was *w* = 0.49 ± 0.21 in the current experiment and *w* = 0.51 ± 0.13 in the rail study in which angles, distances, and lighting conditions were rather different. The similarity of both results suggests that weighting factors of physical and proximal angles are inherent quantities of a person’s visual system.

### Inconsistency Between Perspective Angles and Depth

All subjects walked up and down the room before the experiment and were well aware of its size and that of the bars on the floor. The judged sizes of the perspective angles in the experiment were not consistent with the length of the bars relative to the width between their ends. Assuming a veridical width of 2 m between the frontoparallel bar ends, the mean of all judged angles of 44° is consistent with bar lengths of 2.5 m, which is half of their physical length. Inconsistency between angles and distances was more striking in a previous study in which subjects judged perspective angles of a long and straight railway line (Erkelens, 2015). They estimated the length of the visible rails many hundreds of meters long, while they judged angles between rails as if these had vanishing points at less than 6 m from them. Angles and distances as they are judged are not compatible in a consistent, physical world. The inconsistency is systematic and generic for perspective angles in both physical and depicted scenes. An explanation of the inconsistency is not readily available. The currently popular theories of Helmholtzian and Bayesian inference (Kersten, Mamassian, & Yuille, 2004; Knill & Pouget, 2004; Mamassian, 2006) do not provide an appropriate answer because the combination of perceived angle and distance is physically impossible, and thus has an a priori likelihood of zero. According to these theories, such combinations cannot be perceived. Another explanation is that inconsistencies between angles and distances may arise when these are processed by different and independent neural mechanisms (Foley, 1972). In that case, inconsistencies are the consequence of optimally determining information about each attribute of the world around us (Smeets & Brenner, 2008). This explanation is not satisfactory either because it is not clear from neurophysiology of the visual cortex why neural processing would prefer angles in between the physical and retinal stimuli.

### Practical Implications

We perceive but are not aware of large inconsistencies between perceived perspective angles and distances. Apparently, we do not mind that perspective angles highly underestimate depth. The reason may be that other attributes provide better depth information. However, what happens in conditions when we have to rely on perspective angles because other attributes are not available? In such conditions, distances may seem much shorter than they are. Driving a car on a long straight road at night or in the fog may be examples of such a condition. Seemingly shorter distances are then not hazardous but even advantageous because they may evoke the driver to be more cautious than usual. The situation is different for pilots approaching a runway. A specific type of spatial disorientation, the “black-hole illusion,” may be caused by a severe underestimation of distance. It occurs on approaches to landing at night when the outside view lacks cues to terrain around the lighted runway (Gibb, 2007). Pilots often confidently proceed with a visual approach, relying on information of the perspective borders of the runway. Due to the black-hole illusion, they experience glide path overestimation so that they initiate an inappropriately steep descent. The result is a shallow approach that lies below the correct glide path for obstacle clearance. The black-hole illusion has caused many tragic accidents. Unfortunately, deep understanding of the problem still awaits (Gibb, 2007). The current results indicate the cause of the problem. Currently, experiments are performed in the lab to find better strategies for visual approaches of runways in conditions of poor visibility.
